# Entropic Order Parameters for Categorical Symmetries in 2D-CFT

**DOI:** 10.3390/e26121064

**Published:** 2024-12-06

**Authors:** Javier Molina-Vilaplana, Pablo Saura-Bastida, Germán Sierra

**Affiliations:** 1 Departamento de Automática, Ingeniería Eléctrica y Tecnología Electrónica, Universidad Politécnica de Cartagena, 30202 Cartagena, Spain; pablo.saura@upct.es; 2 Instituto de Física Teórica, CSIC-Universidad Autónoma de Madrid, 28049 Madrid, Spain; german.sierra@csic.es

**Keywords:** relative entropy, categorical symmetries, conformal field theory, symmetry breaking

## Abstract

In this work, we propose an information theoretic order parameter able to characterize the presence and breaking of categorical symmetries in (1+1)-d rational conformal field theories (RCFTs). Specifically, we compute the quantum relative entropy between the ground states of RCFTs representing the critical point of phase transitions between different symmetry-broken phases of theories with categorical symmetries, and their symmetrized versions. We find that, at leading order in the high temperature limit, this relative entropy only depends on the expectation values of the quantum dimensions of the topological operators implementing the categorical symmetry. This dependence suggests that our proposal can be used to characterize the different broken phases of (1+1)-d theories with categorical symmetries.

## 1. Introduction

The last few years have witnessed remarkable developments in the notion of a global symmetry in theoretical physics. The major idea comes from realizing that every symmetry operation can be associated with a topological operator [1]. In this new framework, symmetries are not only captured by a discrete or continuous group, but also by more general mathematical structures such as higher groups and fusion categories. This new viewpoint, known as categorical symmetry, has led to a paradigm shift and fostered new directions in the study of quantum field theories (QFT) (see [2] for a comprehensive and pedagogical review on these developments).

Global symmetries are central to the understanding of physics at low energies. As a fact, they characterize the different phases of a theory in terms of spontaneously symmetry-broken (SSB) phases and the corresponding phase transitions between such phases. For group-like symmetries, this refers to what is known as the Landau paradigm. Due to the extension of the concept of global symmetry represented by categorical symmetries, there has been a recent interest in the study of phases in QFT constrained by these categorical symmetries [3,4]. The aim is thus to generalize the standard Landau theory to a categorical Landau paradigm in which novel phases and phase transitions may arise. In this scenario, for instance, it has been shown that for (1+1)-d gapped phases with categorical symmetries, contrary to the case of the usual group-like symmetries, the different vacua of a gapped phase may be physically distinguishable [3]. This depends on the properties of interfaces between two distinct vacua which are given by the topological line defects of the non-invertible symmetry.

In order to provide new tools to illuminate the role of generalized categorical symmetries in QFT, here, following [5], we propose an order parameter able to characterize the presence and breaking of these symmetries and, thus, the new phases of matter related to those symmetries. This order parameter can be naturally defined using information theoretic quantities and thus can be cast as an entropic order parameter.

Specifically, we study a concrete class of (1+1)-d QFT known as rational conformal field theories (RCFTs), representing the critical point of phase transitions between the different (1+1)-d symmetry-broken phases of theories with categorical symmetries, and the entropic order parameter proposed here allows us to focus on the vacuum states of these theories, which are defined on a flat Euclidean spacetime. Following [5], we use the information theoretic quantity known as the quantum relative entropy, which intuitively, quantifies the difference between two quantum states [6]. It is known that the use of relative entropy in the context of QFT poses several advantages with respect to other entropic quantities, such as the entropy [5,7,8]. Namely, all other information quantities can be derived from it.

The paper is structured as follows: In Section 2, we present a brief overview of the basic material covering RCFTs and topological operators implementing global categorical symmetries. In Section 3, we introduce a generalization of the concept of symmetrization of quantum states for the case of modular fusion categories (MFCs) in RCFTs. Then, following [5], the relative entropy between density matrices and their symmetrizations over an MFC, is proposed as an entropic order parameter. In Section 4, we compute the relative entropy between the ground states of RCFTs representing the critical point of phase transitions between different symmetry-broken phases of theories with categorical symmetries and their symmetrized versions. We find that at leading order, this relative entropy only depends on the expectation values of the TDLs or quantum dimensions of the topological operators implementing the symmetry. Finally, we present our conclusions and perspectives for extensions of this work in Section 5.

## 2. Background

In this section, we review some very basic facts about RCFTs, and topological operators implementing categorical symmetries in these theories. A more comprehensive treatment of these topics can be found in [9].

We consider diagonal RCFTs with a chiral algebra A and its modular tensor category (MTC) C given in terms of simple objects a,b,c,…∈C.

In these theories, topological lines are in one-to-one correspondence with its bulk primaries and are called Verlinde lines. Those act on the bulk primaries as [9],

(1) $$\begin{array}{c} b|\phi_{a}\rangle =\frac{S_{ba}}{S_{1a}}|\phi_{a}\rangle , \end{array}$$

where $S_{ab}$ denotes an element of the unitary *S*-matrix of the RCFTs.

The TDLs a∈C, satisfy the same fusion algebra as that of the bulk primaries, that is

(2) $$\begin{array}{c} a\times b=\sum_{c\in C}N_{ab}^{c}c\;, \end{array}$$

where $N_{ab}^{c}\in Z_{\geq 0}$ are the fusion coefficients given by the Verlinde formula [10]

(3) $$N_{ab}^{c}=\sum_{d\in C}\frac{S_{da}S_{db}S_{dc}^{*}}{S_{1d}}\;.$$

An important property of any TDL b∈C is its quantum dimension $d_{b}$. This is defined as the vacuum expectation value

(4) $$\begin{array}{c} \langle 1|b|1\rangle =\frac{S_{b1}}{S_{11}}\equiv d_{b}\; \end{array}$$

In any unitary CFT with a unique vacuum, $d_{b}\geq 0$ [9].

We consider the torus partition function (with the temporal $S^{1}$ along the vertical axis with length *T* and the spatial $S^{1}$ along the horizontal axis with length *L*) given by
(5)
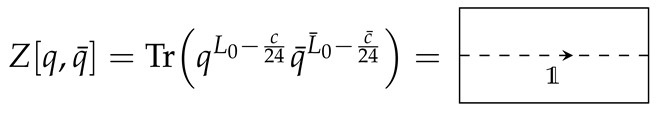

where $q=e^{2\pi i\tau }$, and τ=iT/L, the modular parameter on the torus, so, $q=e^{-2\pi (T/L)}$. The density matrix ρ of the ground state is given thus by

(6) $$\rho =\frac{q^{L_{0}-\frac{c}{24}}{\bar{q}}^{{\bar{L}}_{0}-\frac{\bar{c}}{24}}}{Z[q,\bar{q}]}\;.$$

In this work, we will consider insertions of TDLs a along the spatial $S^{1}$ which can be depicted as follows: (7)
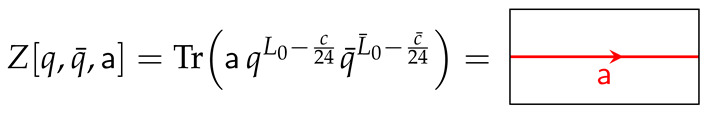

which, from here in advance we simply denote by Z[q,a]. It is important to note that
(8)
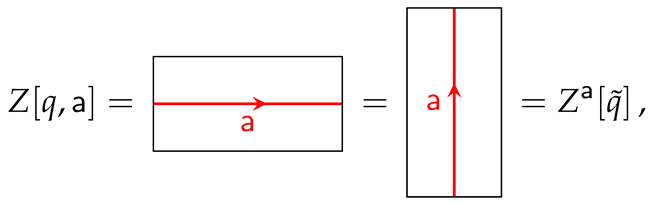

where $\tilde{q}$ is obtained under a modular *S*-transformation $\tau \to -\frac{1}{\tau }$ and that, in the limit in which we work here, L≫T, hence $\tilde{q}\to 0$,

(9) $$\frac{Z[q,a]}{Z[q,1]}∼{\tilde{q}}^{\Delta_{1}^{a}}\to 0\;,$$

where $\Delta_{1}^{a}>0$ is the scaling dimension of the vacuum corresponding to the vacuum of the a-twisted sector and taking $\Delta_{1}^{1}=0$.

Generically, when a topological operator is defined along the whole space at a fixed time, it amounts to a conserved operator that acts on the Hilbert space H. If the topological operator is inserted in the time direction and localized in one of the spatial directions, the topological operator is a defect that modifies the quantization. The modified quantization gives rise to a twisted Hilbert space [11]. We will not consider the later insertions in this work.

## 3. Averages over Categories and Relative Entropy as Entropic Order Parameter

In this section we introduce a generalization of averages over groups for the case of modular fusion categories (MFCs) in RCFTs. We first review the case of Abelian groups and then we introduce the generalization. Then, following [5], the relative entropy between density matrices and their averages over an MFC, is proposed as an entropic order parameter.

### 3.1. Group and Category Averages

In order to fix concepts, let us first recall the symmetrization of ρ as the average over a discrete Abelian group *G* of the transformed density matrix $g\;\rho \;g^{-1}$, that is,

(10) $$\rho_{G}=\frac{1}{\left|G\right|}\sum_{g\in G}\;g\;\rho \;g^{-1},$$

where |G| is the normalized measure of the group, that can be generalized to generic compact Lie groups. Equation (10) implements a map between density matrices known as conditional expectation [5,6]. In essence, this map extracts the portion of ρ that remains unchanged under the action of the group *G*. The density matrix $\rho_{G}$ is by construction symmetric under *G* and has trace one. Note that ρ is symmetric if and only if $\rho =\rho_{G}$. Therefore, comparing the two states ρ and $\rho_{G}$ would lead naturally to probing (spontaneous or explicit) symmetry-breaking [5].

Here, we introduce the average over a category that can be naturally defined as

(11) $$\rho_{C}=\frac{1}{N}\sum_{a\in C}\;a\;\rho \;\bar{a}\;,$$

where $\bar{a}$ is the conjugate of a, that is $a\times \bar{a}=1⊕\ldots$. The constant N is fixed imposing that $\rho_{C}$ is a density matrix,

(12) $$\mathrm{Tr}\rho_{C}=1⟹N=\sum_{a,b\in C}N_{a\bar{a}}^{b}\mathrm{Tr}\left[\rho \;b\right]$$

For group-like invertible symmetries we will have

(13) $$N_{a\bar{a}}^{b}=\delta_{b1}⟹N=\sum_{a\in C}\mathrm{Tr}\left[\rho 1\right]=\left|C\right|$$

and we will recover the standard definition of $\rho_{G}$. However, unlike the case of a group, this map generally does not implement a conditional expectation. Despite this, $\rho_{C}$ in Equation (11) is a well defined quantum state.

To see this, we refer to [12] where the authors show that non-invertible topological lines generally act on local operators through quantum operations or quantum channels, that is, through completely positive maps between density matrices. In this framework, the operation in Equation (11) should be viewed as a combination of such linear operations with conditional expectations corresponding to specific instances of these general class of quantum operation. In addition, we note that due to the positivity of ρ, the expectation value

(14) $$\begin{array}{c} \langle \psi |\rho_{C}|\psi \rangle =\frac{1}{N}\;\sum_{a\in C}\;\langle \psi \;|a\;\rho \;\bar{a}\;|\psi \rangle \geq 0\;, \end{array}$$

and thus $\rho_{C}$ is positive. Finally, the condition that a map preserves total probability thus describing the entirety of an operation’s outcome rather than a specific subset corresponds to trace-preserving completely positive maps, a property ensured by Equation (13). These conditions collectively ensure that $\rho_{C}$ can be used later in the context of relative entropies. This being said, it is important to remark that a systematic study clarifying the extent to which Equation (11) represents the invariant part of the density matrix under the non-invertible symmetry is needed. This point is significant and merits further investigation, which we plan to address in a future work, remarking that it is possible to define conditional expectations for categorical symmetries [13], which are not of the form of Equation (11).

### 3.2. Relative Entropy as an Entropic Order Parameter

The relative entropy between two reduced density matrices ρ,σ is given by

(15) S(ρ||σ)=Trρlogρ−Trρlogσ,

that can be derived from

(16) $$\begin{array}{cc} S(\rho ||\sigma ) & =\lim_{n\to 1}\frac{1}{n-1}\log\left(\frac{\mathrm{Tr}[\rho^{n}]}{\mathrm{Tr}[\rho \;\sigma^{n-1}]}\right)\;. \end{array}$$

Relevant properties of this quantity can be found in [6]. The relative entropy can be operationally interpreted as follows: given σ, the probability *p* of mistaking ρ for σ after *N* well-designed experimental measurements decays with *N* as $p∼e^{-N\;S(\rho ||\sigma )}$, which endorses the relative entropy as an experimentally accessible quantity.

Here, we propose entropic order parameters that allow us to better understand the symmetry breaking of categorical symmetries in 2D CFTs [3]. Following [5], we investigate the behavior of the relative entropy $S(\rho_{G}||\rho )$ (resp. $S(\rho_{C}||\rho )$) between the ground states ρ of the theory and their averaged versions $\rho_{G}$ (resp. $\rho_{C}$). It is expected that $S(\rho_{G}||\rho )$ (resp. $S(\rho_{C}||\rho )$) explicitly depend on the vacuum expectation values of the TDLs implementing the symmetries under consideration [5]. For the group-like invertible symmetries, it is known that the relative entropy vanishes if the state ρ is invariant under the action of symmetry, i.e., ρ is an unbroken symmetry vacuum, and then the two states that the relative entropy compares are identical. Nevertheless, if the group-like symmetry is broken, the relative entropy between these states must be necessarily non-zero. Hence, the usefulness of this concept as an order parameter in those cases.

Our aim here is to extend this program to the case of symmetries given by modular fusion categories in RCFTs.

## 4. Relative Entropy in CFTs with Categorical Symmetries

Here we compute the relative entropy between the category-averaged density matrix $\rho_{C}$ and ρ defined by

(17) $$S(\rho_{C}\left||\rho \right)=\lim_{n\to 1}\;\frac{1}{n-1}\log\;\left[\frac{\mathrm{Tr}\;[\rho_{C}^{n}]}{\mathrm{Tr}\;\left[\rho_{C}\;\rho^{n-1}\right]}\right]\;.$$

We focus first on the term $\mathrm{Tr}\;\left[\rho_{C}\;\rho^{n-1}\right]$. We note that all a∈C are topological and therefore, they are free to move along the Euclidean time direction between the sheets of the replicated manifold under continuous deformations. Indeed, these movements allow to fuse them. With this, one may write the term of interest as

(18) $$\begin{array}{cc} \mathrm{Tr}\;\left[\rho_{C}\;\rho^{n-1}\right] & =\frac{1}{N}\sum_{a\in C}\;\mathrm{Tr}\;\left[a\rho \bar{a}\rho^{n-1}\right]=\frac{1}{N}\sum_{a\in C}\;\mathrm{Tr}\;\left[\rho^{n}a\bar{a}\right]\\ \; & =\frac{1}{N}\sum_{a,b\in C}\;N_{a\bar{a}}^{b}\mathrm{Tr}\;\left[\rho^{n}b\right]=\frac{1}{N}\sum_{a,b,c\in C}\;\frac{{\left(S_{ac}\right)}^{2}}{S_{1c}}S_{bc}\mathrm{Tr}\;\left[\rho^{n}b\right]\\ \; & =\frac{1}{N}\sum_{b,c\in C}\;\left(\sum_{a\in C}{\left(S_{ac}\right)}^{2}\right)\frac{S_{bc}}{S_{1c}}\;\mathrm{Tr}\;\left[\rho^{n}b\right]=\frac{1}{N}\sum_{b,c\in C}\;\frac{S_{bc}}{S_{1c}}\;\mathrm{Tr}\;\left[\rho^{n}b\right]\;, \end{array}$$

where we have used the Verlinde formula and the fact that $\left(\sum_{a\in C}{\left(S_{ab}\right)}^{2}\right)=1$.

As we are interested in exploring the asymptotic limit $\tilde{q}\to 0$, it is interesting to write the above result as

(19) $$\begin{array}{cc} \mathrm{Tr}\;\left(\rho_{C}\rho^{n-1}\right) & =\frac{1}{N}\left(\sum_{c\in C}\mathrm{Tr}\;\left[\rho^{n}\right]+\sum_{b\neq 1,c\in C}\;\frac{S_{bc}}{S_{1c}}\;\mathrm{Tr}\;\left[\rho^{n}b\right]\right)\\ \; & =\frac{\left|C\right|}{N}\mathrm{Tr}\;\left[\rho^{n}\right]+\frac{1}{N}\sum_{b\neq 1,c\in C}\;\frac{S_{bc}}{S_{1c}}\;\mathrm{Tr}\;\left[\rho^{n}b\right]\\ \; & =\frac{\left|C\right|}{N}\mathrm{Tr}\;\left[\rho^{n}\right]\left(1+\frac{1}{\left|C\right|}\sum_{b\neq 1,c\in C}\;\frac{S_{bc}}{S_{1c}}\;\frac{\mathrm{Tr}\;\left[\rho^{n}b\right]}{\mathrm{Tr}\;\left[\rho^{n}\right]}\right)\;, \end{array}$$

because, as commented above, in the limit $\tilde{q}\to 0$,

(20) $$\frac{\mathrm{Tr}\;\left[\rho^{n}b\right]}{\mathrm{Tr}\;\left[\rho^{n}\right]}\approx {\tilde{q}}^{\Delta_{b}^{1}/n}\to 0\;,$$

with $\Delta_{b}^{1}$ the scaling dimension of the vacuum corresponding to the vacuum of the b-twisted sector. Therefore, the quantity

(21) $$\Delta_{n}\left[C\right]=\frac{1}{\left|C\right|}\sum_{b\neq 1,c\in C}\;\frac{S_{bc}}{S_{1c}}\;\frac{\mathrm{Tr}\;\left[\rho^{n}b\right]}{\mathrm{Tr}\;\left[\rho^{n}\right]}\;,$$

is subleading and only appears in the fusion category case, as in the group like case, b can only be the identity in $N_{a\bar{a}}^{b}$ appearing in (18), that is, $\Delta_{n}\left[C\right]$ identically vanishes for the group-like case. Now, by writing

(22) $$\mathrm{Tr}\;\left(\rho_{C}\rho^{n-1}\right)=\mathrm{Tr}\;\left[\rho^{n}\right]\left[\frac{\left|C\right|}{N}\;\left(1+\Delta_{n}\left[C\right]\right)\;\right]\;,$$

one may express the relative entropy as

(23) $$\begin{array}{cc} S(\rho_{C}||\rho ) & =\lim_{n\to 1}\;\frac{1}{n-1}\log\;\left[\frac{\mathrm{Tr}\;\left[\rho_{C}^{n}\right]}{\mathrm{Tr}\;\left[\rho^{n}\right]\left[\frac{\left|C\right|}{N}\;\left(1+\Delta_{n}\left[C\right]\right)\;\right]}\right]\\ \; & =\lim_{n\to 1}\;\frac{1}{n-1}\left(\log\;\frac{\mathrm{Tr}\;\left[\rho_{C}^{n}\right]}{\mathrm{Tr}\;\left[\rho^{n}\right]}-\log\left[\frac{\left|C\right|}{N}\;\left(1+\Delta_{n}\left[C\right]\right)\right]\right)\\ \; & =\lim_{n\to 1}\left(-\Delta S_{n}+\frac{1}{1-n}\;\log\left[\frac{\left|C\right|}{N}\;\left(1+\Delta_{n}\left[C\right]\right)\right]\right)\;. \end{array}$$

Noting that

(24) $$\begin{array}{cc} N & =\sum_{a,b\in C}N_{aa}^{b}\mathrm{Tr}\left[\rho \;b\right]=\sum_{a,b\in C}\sum_{c}\frac{S_{ac}S_{ac}S_{bc}}{S_{1c}}\mathrm{Tr}\left[\rho \;b\right]\\ \; & =\sum_{b,c}\frac{S_{bc}}{S_{1c}}\;\mathrm{Tr}\left[\rho b\right]=\left|C\right|\;\mathrm{Tr}\left[\rho \right]+\sum_{b\neq 1,c}\frac{S_{bc}}{S_{1c}}\;\mathrm{Tr}\left[\rho b\right]\\ \; & =|C|\;\mathrm{Tr}\left[\rho \right]\left(1+\frac{1}{\left|C\right|}\sum_{b\neq 1,c}\frac{S_{bc}}{S_{1c}}\;\frac{\mathrm{Tr}[\rho b]}{\mathrm{Tr}[\rho ]}\right)=\left|C\right|\;\left(1+\Delta_{1}\left[C\right]\right)\;, \end{array}$$

where we have used that, for diagonal RCFTs, $\bar{a}=a$ and we have taken the definition of $\Delta_{1}\left[C\right]$ from (26). One may finally write

(25) $$S(\rho_{C}\left||\rho \right)=\lim_{n\to 1}\left(-\Delta S_{n}+\frac{1}{1-n}\;\log\;\frac{\left(1+\Delta_{n}\left[C\right]\right)}{\left(1+\Delta_{1}\left[C\right]\right)}\right)\;,$$

where $\Delta S_{n}$ is given by,

(26) $$\Delta S_{n}=\frac{1}{1-n}\log\frac{\mathrm{Tr}[\rho_{C}^{n}]}{\mathrm{Tr}[\rho^{n}]}\;.$$

This quantity, when restricted to a subsystem *A* of the theory, is known as entanglement asymmetry, quantifying the degree of symmetry breaking in a region of an extended quantum system [14,15].

We note that while for the group-like case, where $\Delta_{n}\left[C\right]=0$ exactly vanishes and thus, $S(\rho_{A,C}\left|\right|\rho_{A})=-\Delta S_{1}$, for the category case, at leading order in $\tilde{q}\to 0$ we have

(27) $$S(\rho_{C}\left||\rho \right)∼-\Delta S_{1}\;.$$

Using the results above, we are now prompted to compute $\Delta S_{n}$. Therefore, by inserting (11) into (26), one obtains

(28) $$\Delta S_{n}=\frac{1}{1-n}\log\frac{1}{N^{n}}\sum_{a_{1},\ldots ,a_{n}\in C}\frac{\mathrm{Tr}\left[\rho \;{\bar{a}}_{1}a_{2}\rho {\bar{a}}_{2}\ldots {\bar{a}}_{n-1}a_{n}\rho {\bar{a}}_{n}a_{1}\right]}{\mathrm{Tr}[\rho^{n}]}\;.$$

With this, the multi-charged moments can be defined as

(29) $$Z\left[q^{n},a\right]=\mathrm{Tr}\left[\rho \;{\bar{a}}_{1}a_{2}\rho {\bar{a}}_{2}\ldots {\bar{a}}_{n-1}a_{n}\rho {\bar{a}}_{n}a_{1}\right]$$

where $a\in C^{n}$ is the *n*-tuple defined by $a=(a_{1},\ldots ,a_{n})$, and graphically reads as
(30)
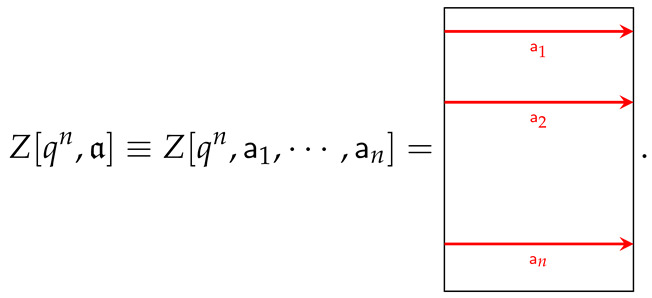


Given this, now Equation (29) can be written as

(31) $$\Delta S_{n}=\frac{1}{1-n}\log\frac{1}{N^{n}}\sum_{a\in C^{n}}\frac{Z[q^{n},a]}{Z[q^{n}]}\;.$$

As commented above, the TDL a∈C are free to move up and down along the (Euclidean) time direction of the replicated manifold under continuous transformations, and this allows to fuse them. Using this again, one can write,

(32) $$Z\left[q^{n},a\right]=\mathrm{Tr}\left[\rho^{n}{\bar{a}}_{1}a_{2}{\bar{a}}_{2}\ldots {\bar{a}}_{n-1}a_{n}{\bar{a}}_{n}a_{1}\right]\;.$$

Now, one may use the fusion algebra $a\times b=\sum_{c}N_{ab}^{c}c$ to, assuming that $\bar{a}=a,\;\forall a\in C$, write,

(33) $$a_{1}a_{2}a_{2}a_{3}a_{3}\ldots a_{n-1}a_{n-1}a_{n}a_{n}a_{1}=\sum_{b^{\prime }s,c^{\prime }s}N_{a_{1}a_{2}}^{b_{1}}N_{b_{1}a_{2}}^{c_{1}}N_{c_{1}a_{3}}^{b_{2}}N_{b_{2}a_{3}}^{c_{2}}N_{c_{2}a_{4}}^{b_{3}}N_{b_{3}a_{4}}^{c_{3}}\ldots N_{c_{n-2}a_{n}}^{b_{n-1}}N_{b_{n-1}a_{n}}^{c_{n-1}}N_{c_{n-1}a_{1}}^{d}\;d$$

Using the Verlinde formula (Equation (3)) and general properties of the *S*-matrix, we obtain (see Appendix A)

(34) $$\sum_{a}Z\left[q^{n},a\right]=\sum_{b\;\in C}\sum_{c\;\in C}\frac{1}{S_{1c}^{2n-1}}\;S_{bc}\;\mathrm{Tr}\left[\rho^{n}\;b\right]\;,$$

that can be represented as
(35)
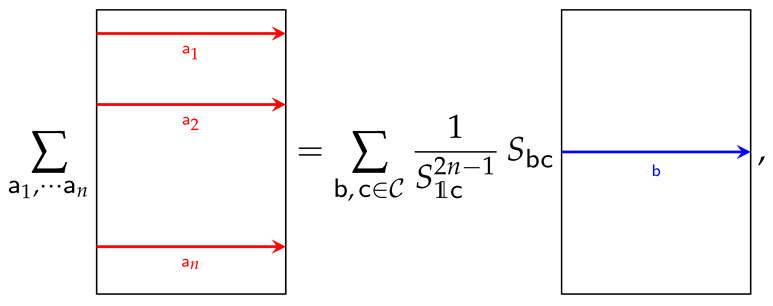


Finally, $\Delta S_{n}$ in (26) can be written as

(36) $$\begin{array}{c} \Delta S_{n}=\frac{1}{1-n}\log\frac{1}{\mathrm{Tr}[\rho^{n}]}\frac{\sum_{b,c}\frac{1}{S_{1c}^{2n-1}}\;S_{bc}\;\mathrm{Tr}\left[\rho^{n}b\right]}{{\left(\sum_{b,c}\frac{S_{bc}}{S_{1c}}\mathrm{Tr}\left[\rho b\right]\right)}^{n}}\;. \end{array}$$

We provide here the asymptotic limit $\tilde{q}\to 0$ for $\Delta S_{n}$ in Equation (36). This amounts to taking only the terms related to b=1 in the sums above, from which one can derive that

(37) $$\Delta S_{n}∼\frac{1}{1-n}\log\frac{1}{\mathrm{Tr}[\rho^{n}]}\frac{\sum_{c}\frac{1}{S_{1c}^{2(n-1)}}\mathrm{Tr}\left[\rho^{n}\right]}{{\left(\sum_{c}\mathrm{Tr}\left[\rho \;1\right]\right)}^{n}}=\frac{1}{1-n}\log\frac{1}{{\left|C\right|}^{n}}\sum_{c\in C}S_{1c}^{2(1-n)}\;.$$

In limit n→1 one finds

(38) $$\Delta S_{1}=\log\left|C\right|+\frac{2}{\left|C\right|}\sum_{c\in C}\log S_{1c}$$

As it is said above, it would be expected that the relative entropy is related to the vacuum expectation values of the TDL implementing the symmetry under consideration. To see this, let us recall the definition of quantum dimension,

(39) $$d_{c}=\frac{S_{1c}}{S_{11}}$$

so we can write (38) as

(40) $$\Delta S_{1}=\log\left|C\right|+2\log S_{11}+\frac{2}{\left|C\right|}\sum_{c\in C}\log d_{c}$$

On the other hand

(41) $$1=\sum_{c\in C}S_{1c}S_{1c}=S_{11}^{2}\sum_{c\in C}d_{c}^{2}$$

which implies

(42) $$S_{11}=\frac{1}{D},\;D=\sqrt{\sum_{c\in C}d_{c}^{2}}$$

where D is the total quantum dimension of the modular tensor category. Replacing (42) into (40) gives,

(43) $$\Delta S_{1}=\log\left|C\right|-2\log D+\frac{2}{\left|C\right|}\sum_{c\in C}\log d_{c}\;.$$

Finally, we express $S(\rho_{C}||\rho )$ in a more illustrative form by observing that the equation above can be written as

(44) $$-\Delta S_{1}=\log\left(\frac{1}{\left|C\right|}\sum_{a\in C}d_{a}^{2}\right)-\frac{1}{\left|C\right|}\sum_{a\in C}\log d_{a}^{2}\;,$$

and therefore, according to our result in Equation (26), at leading order for categories, and exactly for groups,

(45) $$S(\rho_{C}\left||\rho \right)=\log\left(\frac{1}{\left|C\right|}\sum_{a\in C}d_{a}^{2}\right)-\frac{1}{\left|C\right|}\sum_{a\in C}\log d_{a}^{2}\;,$$

which implies that $S(\rho_{C}||\rho )\geq 0$ always. To see where this property comes from, we may rewrite the relative entropy as:

(46) $$S(\rho_{C}\left||\rho \right)=\log\frac{\left(\sum_{a}d_{a}^{2}\right)/\left|C\right|}{\sqrt[{\left|C\right|}]{\left(\prod_{a}d_{a}^{2}\right)}}\;.$$

We notice that this is the quotient between the arithmetic mean and the geometric mean of $d_{a}^{2}$. As $d_{a}^{2}\in R^{+}$, we can invoke the AM–GM inequality [16], resulting in the desired property $S(\rho_{C}||\rho )\geq 0$. Moreover, the inequality only saturates if all the elements in the mean values are equal to each other. In our case, we always must have an element satifying $d_{1}=1$, i.e., the identity. This means that $S(\rho_{c}||\rho )=0$ if and only if $d_{a}=1\;\;\forall a$, that is, just for group-like symmetries. This result is interesting as it shows that,

1. At leading order, the relative entropy $S(\rho_{C}||\rho )$ only depends on the expectation values of the TDLs or quantum dimensions.
2. As commented above, for groups, in which every symmetry operator is implemented by a TDL a with $d_{a}=1$, $S(\rho_{G}||\rho )=0$. As an example, for the $G=Z_{2}$ group, we have two invertible TDLs {1,η} with η×η=1 and,
  (47) $$d_{1}=1,\;d_{\eta }=1$$
  Thus,
  (48) $$S(\rho_{G}\left||\rho \right)=-\Delta S_{1}=0\;.$$
3. For systems with non-invertible symmetries represented by a fusion category C, there are some TDL’s a with $d_{a}>1$, and therefore, $S(\rho_{C}||\rho )>0$. As an example, for the Fibonacci category $C_{\mathrm{Fib}}$ we have two TDLs {1,W} with W×W=1+W and,
  (49) $$d_{1}=1,\;d_{W}=\phi$$
  where $\phi =(1+\sqrt{5})/2$. Thus,
  (50) $$S(\rho_{C}\left||\rho \right)=-\Delta S_{1}=0.111572\ldots$$
4. At leading order, the relative entropy $S(\rho_{C}||\rho )$ essentially counts the number of invariant configurations of the replica partition functions under the action of TDLs insertions, that is, for how many configurations $a\in C^{n}$, $Z\left[q^{n},a\right]∼Z\left[q^{n},1\right]$. It has been shown that in spontaneously broken phases, the action of TDLs implementing symmetries on the vacua of the theory may be different depending on the phase [3,4]. In our setting, this would lead to a different counting on the configurations satisfying $Z\left[q^{n},a\right]∼Z\left[q^{n},1\right]$. This suggest that $S(\rho_{C}||\rho )$ may characterize distinct phases associated to the SSB of categorical-symmetries.

## 5. Conclusions and Outlook

We have studied the relative entropy between the ground states of RCFTs representing the critical point of phase transitions between different symmetry-broken phases of theories with categorical symmetries and their symmetrized versions.

We find that at leading order, this relative entropy only depends on the expectation values of the TDLs or quantum dimensions of the topological operators implementing symmetry. This dependence is such that, for group-like symmetries in which all the quantum dimensions implementing the symmetry are equal to one, this order parameter vanishes identically. On the other hand, in the case of categorical symmetries, we show that this order parameter is not zero. Our results suggest that our proposal can be used to characterize different broken phases of 2D theories with categorical symmetries.

As said above, as the relative entropy is an experimentally accessible quantity, we note that, in extended quantum systems such as the theories addressed here, taking a measure is concomitant of considering a specific subsystem. In this situation, it is thus sensible to extend our ideas by using tools from the theory of entanglement. The entanglement asymmetry, commented above, has been recently introduced as a measure of symmetry breaking at a subsystem level. It is worth investigating if our entropic order parameter for categorical symmetries can be extended to this framework, following the ideas in [17].

## Data Availability

Data is contained within the article.

## Appendix A

In this appendix, we show how to derive Equation (34) in the main text. Let us focus momentarily in the case n=3 where

(A1) $$a_{1}a_{2}a_{2}a_{3}a_{3}a_{1}=\sum_{b_{1},b_{2},c_{1},c_{2}}N_{a_{1}a_{2}}^{b_{1}}N_{b_{1}a_{2}}^{c_{1}}N_{c_{1}a_{3}}^{b_{2}}N_{b_{2}a_{3}}^{c_{2}}N_{c_{2}a_{1}}^{d}\;d$$

Using the Verlinde formula

(A2) $$N_{ij}^{k}=\sum_{m}\frac{S_{im}S_{jm}S_{km}}{S_{1m}}$$

we obtain

(A3) $$\sum_{j,k}N_{ij}^{k}N_{kj}^{l}=\sum_{j,k}\sum_{m,n}\frac{S_{im}S_{jm}S_{km}}{S_{1m}}\frac{S_{kn}S_{jn}S_{lm}}{S_{1l}}=\sum_{m}\frac{S_{im}S_{lm}}{S_{1m}^{2}}$$

where we used that $\sum_{j}S_{ij}S_{jk}=\delta_{ik}$ and $S_{ij}=S_{ji}$. The sum over the labels in (A1) gives

(A4) $$\begin{array}{ccc} \sum_{a_{1},a_{2},a_{3}}a_{1}a_{2}a_{2}a_{3}a_{3}a_{1} & = & \sum_{a_{1},c_{1},c_{2}}\left(\sum_{a_{2},b_{1}}N_{a_{1}a_{2}}^{b_{1}}N_{b_{1}a_{2}}^{c_{1}}\right)\left(\sum_{a_{3},b_{2}}N_{c_{1}a_{3}}^{b_{2}}N_{b_{2}a_{3}}^{c_{2}}\right)N_{c_{2}a_{1}}^{d}\;d\\ & = & \sum_{a_{1},c_{1},c_{2}}\sum_{m_{1},m_{2}}\frac{S_{a_{1}m_{1}}S_{c_{1}m_{1}}}{S_{1m_{1}}^{2}}\frac{S_{c_{1}m_{2}}S_{c_{2}m_{2}}}{S_{1m_{2}}^{2}}N_{c_{2}a_{1}}^{d}\;d\\ & = & \sum_{a_{1},c_{1},c_{2}}\sum_{m_{1},m_{2},m_{3}}\frac{S_{a_{1}m_{1}}S_{c_{1}m_{1}}}{S_{1m_{1}}^{2}}\frac{S_{c_{1}m_{2}}S_{c_{2}m_{2}}}{S_{1m_{2}}^{2}}\frac{S_{c_{2}m_{3}}S_{a_{1}m_{3}}S_{dm_{3}}}{S_{1m_{3}}}\;d\\ & = & \sum_{m_{1},m_{2},m_{3}}\frac{\delta_{m_{1}m_{2}}\delta_{m_{2}m_{3}}\delta_{m_{3}m_{1}}S_{dm_{3}}}{S_{1m_{1}}^{2}S_{1m_{2}}^{2}S_{1m_{3}}}\;d=\sum_{m}\frac{S_{dm}}{S_{1m}^{5}}\;d \end{array}$$

For arbitrary *n* it is easy to find the general results which reads as,

(A5) $$\sum_{a_{1},\ldots ,a_{n}}a_{1}a_{2}a_{2}\ldots a_{n-1}a_{n}a_{n}a_{1}=\sum_{m}\frac{S_{dm}}{S_{1m}^{2n-1}}\;d\;,$$

and hece

(A6) $$\sum_{a}Z\left[q^{n},a\right]=\sum_{d}\sum_{m}\frac{S_{dm}}{S_{1m}^{2n-1}}\mathrm{Tr}\left(\rho^{n}d\right)\;.$$
